# Editorial: Exploration of novel approaches to determine and improve the microbiological quality of food products

**DOI:** 10.3389/fmicb.2022.1108305

**Published:** 2023-01-06

**Authors:** Slim Smaoui, Abdo Hassoun, Atika Meklat

**Affiliations:** ^1^Center of Biotechnology of Sfax, Sfax, Tunisia; ^2^Sustainable AgriFoodtech Innovation & Research (Safir), Arras, France; ^3^École Normale Supérieure de Kouba, Algiers, Algeria

**Keywords:** natural antimicrobials, new microbiological approaches, modern analytical methods, food safety, food quality, foodborne pathogens, food industry

As editors of this Research Topic, it was our pleasure to examine a wide range of attractive articles and reviews within the field of food microbiology. In this editorial, we outline the major results and perspectives detailed within each of the accepted manuscripts. This Research Topic is composed of two review articles and four original research articles.

In recent years, the omic sciences have contributed significantly to the development of methods for understanding the impact of various ingredients and foods on the human body on a molecular level. Tools that have recently been used to investigate numerous components and metabolites that can be valuable in the context of human consumption, and in particular bacteriocins, are discussed in the review presented by Dutta et al.. This review examines various current methods, including proteomics, transcriptomics, and metabolomics, for the overall enhancement of the quality of food products *via* bacteriocin application. The matrix associated with food products requires the use of sophisticated technologies that aid with the extraction of the large amount of information required for the amelioration of food products. This paper offers an overview of bacteriocins in terms of their structure, chemical composition, metabolomics, and their practical use in food preservation.

The review by Ghosh et al. presents an in-depth discussion of the application of clustered regularly interspaced short palindromic repeats interference (CRISPRi) in food preservation and manufacturing. In particular, CRISPRi-facilitated silencing of genes encoding regulatory proteins associated with biofilm production is considered to be a reliable approach to editing gene networks in various biofilm-forming bacteria, either *via* inactivation of biofilm-forming genes or *via* integration into the host genome of genes corresponding to antibiotic resistance or fluorescent markers.

While the cultivation techniques, breeding techniques, and physiology of *Volvariella volvacea*, a mushroom that is industrially grown in many tropical and subtropical regions, have been thoroughly investigated, little is known about the characteristics of degenerated strains. Zhao et al. investigated the results of 20 months of continuous subculturing of mycelia to obtain degenerated *V. volvacea* strains S1-S20. In this context, the authors measured the characteristics of the mycelia and fruiting bodies, the reactive oxygen species (ROS) content, and enzymatic activities in order to explore the physiological changes occurring in subcultured strains. Analysis of ROS content, lignocellulase and antioxidant enzyme activities, and gene expression *via* reverse transcription polymerase chain reaction (RT-PCR) indicated that strain degradation in this fungal species was accompanied by a reduction in the activity of substrate-degrading enzymes and an extreme accumulation of ROS. The latter further led to aging of the organism during successive mycelial subculturing and may have been responsible for strain degradation in *V*. *volvacea*.

Remaining on the subject of antioxidant properties, Yan et al. discuss and analyze the current state of knowledge on carotenoids, a group of natural pigments acting as precursors to vitamin A, produced by *Sporobolomyces pararoseus*. It should be noted that Geranylgeranyl diphosphate synthase (GGPPS) is considered to be a key enzyme in the carotenoid biosynthesis pathway. The authors describe the cloning of a cDNA copy of the GGPPS protein-encoding gene crtE from *S. pararoseus* NGR. The crtE full-length genomic DNA and cDNA are 1,722 and 1,134 bp, respectively, which consist of 9 exons and 8 introns. This gene encodes a protein of 377 amino acids with a predicted molecular mass of 42.59 kDa and a PI of 5.66. Identification of the crtE gene encoding a functional GGPPS was achieved using heterologous complementation detection in *Escherichia coli*. *In vitro* enzymatic activity experiments showed that CrtE utilizes farnesyl diphosphate as an allylic substrate for the condensation reaction with isopentenyl diphosphate (IPP), generating more of the unique product GGPP compared to other allylic substrates. The predicted crtE 3D model is analyzed in comparison with yeast GGPPS. The condensation reaction occurs in the cavity of the subunit, and three bulky amino acids (Tyr110, Phe111, and His141) below the cavity prevent further extension of the product. The authors' findings provide a new source of genes for carotenoid genetic engineering. Furthermore, these findings improve the understanding of the synthetic pathway of carotenoids in *S. pararoseus* NGR and unveil a new gene target for further improvement of carotenoid production *via* genetic engineering.

From the perspective of measurability and effectiveness based on fingerprints, machine learning, and network pharmacology, Li et al. report on an attempt to screen for potential biomarkers in various aspects of *Wolfiporia cocos*. This mushroom is extensively utilized in traditional Chinese medicine and as a dietary supplement. Using HPLC, the authors identify three components, *viz*. dehydrotrametenolic acid, poricoic acid A, and pachymic acid, as key potential biomarkers. In addition, their integrated analysis suggests that these molecules are probable biomarkers for *Poria* and *Poriae* cutis. Interestingly, the proposed approach represents an original strategy for the study of potential biomarkers, with the potential to provide insight into the clinical applications and reasonable development and utilization of *Poria* and *Poriae* cutis.

In the investigation by Zhu et al., the authors select two types of “green-covering” Tuqu (TQ) with a crucial function in generating light-aroma-type Baijiu (LATB), namely TQ with a non-red heart (NRH) and with a red heart (RH), for an exploration of the diversity of the microbial community through high-throughput sequencing (HTS). Their results exposed dissimilarity in the microbial communities of different types of TQ and led to the isolation of two *Monascus* species. Remarkably, HPLC did not identify the presence of citrinin, demonstrating that *Monascus* isolated from TQ does not represent a safety risk. Throughout the fermentation process, γ-aminobutyric acid content was found to be higher in the fermented grains of RH than in those of NRH. Through use of HTS and gas chromatography–flame ionization detection (GC-FID), the effects of the fortified strain *Monascus* on fungal microbial composition in TQ and flavor substances in Baijiu were examined, yielding LATB with varied flavor profiles. In addition, *Monascus* was found to exert an inhibitory effect on the growth of *Saccharomyces* and *Aspergillus*. The reported findings provide specialized fungal resources for screening of superior strains and improving the quality of TQ and the original liquor.

## Author contributions

AH and AM were involved in draft preparation. SS was involved in supervision and project administration, editing, and final version writing. All authors contributed to the article and approved the submitted version.

